# Role of MicroRNA Modulation in the Interferon-α/Ribavirin Suppression of HIV-1 *In Vivo*

**DOI:** 10.1371/journal.pone.0109220

**Published:** 2014-10-02

**Authors:** Mohamed Abdel-Mohsen, Xutao Deng, Ali Danesh, Teri Liegler, Evan S. Jacobs, Andri Rauch, Bruno Ledergerber, Philip J. Norris, Huldrych F. Günthard, Joseph K. Wong, Satish K. Pillai

**Affiliations:** 1 Department of Medicine, University of California San Francisco, San Francisco, California, United States of America; 2 Blood Systems Research Institute, San Francisco, California, United States of America; 3 Department of Infectious Diseases, Bern University Hospital and University of Bern, Bern, Switzerland; 4 Division of Infectious Diseases and Hospital Epidemiology, University Hospital Zurich, University of Zurich, Zurich, Switzerland; 5 Department of Laboratory Medicine, University of California San Francisco, San Francisco, California, United States of America; 6 Department of Medicine, San Francisco Veterans Affairs Medical Center, San Francisco, California, United States of America; Temple University School of Medicine, United States of America

## Abstract

**Background:**

Interferon-α (IFN-α) treatment suppresses HIV-1 viremia and reduces the size of the HIV-1 latent reservoir. Therefore, investigation of the molecular and immunologic effects of IFN-α may provide insights that contribute to the development of novel prophylactic, therapeutic and curative strategies for HIV-1 infection. In this study, we hypothesized that microRNAs (miRNAs) contribute to the IFN-α-mediated suppression of HIV-1. To inform the development of novel miRNA-based antiretroviral strategies, we investigated the effects of exogenous IFN-α treatment on global miRNA expression profile, HIV-1 viremia, and potential regulatory networks between miRNAs and cell-intrinsic anti-HIV-1 host factors *in vivo*.

**Methods:**

Global miRNA expression was examined in longitudinal PBMC samples obtained from seven HIV/HCV-coinfected, antiretroviral therapy-naïve individuals before, during, and after pegylated interferon-α/ribavirin therapy (IFN-α/RBV). We implemented novel hybrid computational-empirical approaches to characterize regulatory networks between miRNAs and anti-HIV-1 host restriction factors.

**Results:**

miR-422a was the only miRNA significantly modulated by IFN-α/RBV *in vivo* (p<0.0001, paired t test; FDR<0.037). Our interactome mapping revealed extensive regulatory involvement of miR-422a in p53-dependent apoptotic and pyroptotic pathways. Based on sequence homology and inverse expression relationships, 29 unique miRNAs may regulate anti-HIV-1 restriction factor expression *in vivo*.

**Conclusions:**

The specific reduction of miR-422a is associated with exogenous IFN-α treatment, and likely contributes to the IFN-α suppression of HIV-1 through the enhancement of anti-HIV-1 restriction factor expression and regulation of genes involved in programmed cell death. Moreover, our regulatory network analysis presents additional candidate miRNAs that may be targeted to enhance anti-HIV-1 restriction factor expression *in vivo*.

## Introduction

Induction of interferon-α (IFN-α) expression is a critical first step in the defense against a range of viral pathogens [1], [2]. Several studies have demonstrated that IFN-α treatment potently suppresses HIV-1 viremia in chronically infected individuals [3]–[6]. A provocative recent report demonstrated that IFN-α treatment results in sustained viral suppression in the absence of antiretroviral therapy (ART) and significant reduction in the size of the HIV-1 reservoir in chronically-infected individuals [7], [8]. A related analysis of the effects of IFN-α/ribavirin therapy on the HIV-1 latent reservoir in HIV/HCV-coinfected individuals reported a similar, significant reduction in reservoir size [9]. These studies collectively demonstrate that IFN-α molecular pathways may be exploited to attack the HIV-1 latent reservoir and achieve HIV-1 eradication.

In contrast to exogenous IFN-α treatment, endogenous IFN-α production and associated gene expression patterns are curiously often associated with rapid HIV-1 disease progression, high viral load and persistent inflammation rather than beneficial disease outcomes [10], [11]. This paradox is mirrored in recent studies of LCMV infection suggesting that IFN-α is associated with both beneficial and detrimental disease outcomes, and disease progression is governed by the overall balance between the various, diverse effects of type I interferon [12], [13]. Focused analyses of IFN-α molecular pathways *in vivo* may allow us to identify specific mechanisms underlying the beneficial effects of IFN-α treatment on the control and clearance of viral infection. IFN-α treatment has been previously associated with an increase in perforin and granzyme A expression by natural killer (NK) cells in HIV-1-infected individuals, suggesting that enhanced NK-mediated anti-HIV-1 cytolytic activity may contribute to viral suppression [14]. Our group recently published data suggesting that several host restriction factors including BST-2/tetherin and members of the tripartite motif (TRIM) and APOBEC3 families play critical roles in the interferon-mediated suppression of HIV-1 viremia in chronically-infected individuals [15], [16] and in the control of HIV-1 *in vivo* in the absence of antiretroviral therapy (ART) [17]. In this study, we hypothesize that microRNAs (miRNAs) contribute to the IFN-α-mediated suppression of HIV-1 by repressing HIV-1 protein translation directly, or by regulating the gene expression of host factors affecting HIV-1 replication and persistence *in vivo*.

miRNAs are a class of small non-protein-coding RNAs (approximately 22 nucleotides in length) that pair with specific “target” messenger RNAs (mRNAs) and play a significant role in regulating gene expression by binding to mRNAs, thereby repressing translation or degrading the mRNA altogether [18]. Solitary miRNAs often regulate expression of multiple genes with related functions; therefore, changes in expression levels of a single miRNA can broadly affect a gene network and modify complex biological processes [18]. miRNAs play a pivotal role in many biological processes, including cellular differentiation and proliferation [19]. Aberrant miRNA levels are associated with a number of disease states including several types of cancer, in which miRNAs can act as tumor suppressors and oncogenes [20].

miRNAs of viral and host origin may influence host-virus interaction by acting as direct modulators of viral replication, as factors affecting viral susceptibility, and as indirect modulators of cellular genes that influence viral propagation [21]–[23]. In the context of HIV-1 infection, a main challenge is to determine the specific roles of the expanding inventory of human miRNAs in HIV-1 pathogenesis, including the functional consequences of miRNA-mRNA interactions [24]. Human miR-28, miR-125b, miR-150, miR-223, and miR-382 target the 3′ UTR of HIV-1 transcripts, interfering with HIV-1 accessory gene expression potentially shifting productive infection into latency in resting CD4+ T lymphocytes [25]. The difference in expression levels of several anti-HIV-1 miRNAs in monocytes and macrophages correlates with cellular permissibility to HIV-1 infection *in vitro* [26]. A recent report suggests that miR-148 regulates the expression of HLA-C at the host cell surface, and this regulatory activity is correlated with control of HIV-1 replication [27]. Taken together, these observations suggest that studying natural expression levels of pro- and anti-HIV-1 miRNAs may prove valuable in understanding susceptibility to infection, and miRNA manipulation may constitute a promising anti-HIV strategy in the future. Recent data suggest that type I interferon modulates cellular miRNA profile as an antiviral mechanism against hepatitis C virus [28]. The relevance of miRNA to the potent IFN-α-mediated suppression of HIV-1, however, remains to be addressed and is the focus of this study.

IFN-α monotherapy is not typically administered to HIV-1-monoinfected individuals. Combination therapy with pegylated IFN-α and ribavirin (IFN-α/RBV) is commonly used to treat HCV infection [4]. In this study, we analyzed longitudinal clinical specimens from IFN-α/RBV-treated, ART-naive HIV/HCV-coinfected individuals to evaluate the role of miRNAs in the suppression of HIV-1 by IFN-α *in vivo*.

## Methods

### Subjects and specimen processing

Longitudinal samples were collected from seven HIV/HCV-coinfected individuals enrolled in the Swiss HIV Cohort Study ([SHCS], www.shcs.ch) [29] who underwent IFN-α/RBV treatment (Table S1). All subjects had PBMC samples available before, during and after IFN-α/RBV treatment (a post-treatment sample was not available for Subject A), were ART-naïve, and had detectable HIV-1 RNA at baseline. The same collection of samples was analyzed in two recent publications from our group that characterized the role of retroviral restriction factors in the IFN-α-mediated suppression of HIV-1 *in vivo* [15], [16], and gene expression data from these prior studies were included in miRNA-mRNA network analyses presented in this report. The research was approved by the institutional review boards at each of the Swiss HIV Cohort Study sites where samples were collected: University Hospital Basel, University Hospital Bern, University Hospital Zurich, and Canton Hospital, St. Gallen. All human participants gave written informed consent.

### Cellular microRNA expression profiling

Total RNA was extracted from PBMC using TRIzol reagent (Invitrogen). miRNA expression was determined by applying the Megaplex Pools protocol (Applied Biosystems). 300 ng of RNA from PBMCs of the seven patients (pre, during, and post time points) was reverse transcribed using the TaqMan MicroRNA reverse transcription kit in combination with the Megaplex RT Primers Pool A that allows the analysis of 377 human miRNAs which represent the most rigorously studied human miRNAs or Megaplex RT Primers Pool B that allows the analysis of 377 newly discovered human miRNAs and endogenous controls. The following cycling conditions were used: 40 cycles at 16°C for 2 minutes, 42°C for 1 minute and 50°C for 1 second followed by 1 step at 85°C for 5 minutes. Reverse transcription was followed by a preamplification of the miRNA cDNA target (2.5 µL) using TaqMan PreAmp Master Mix kit and Megaplex PreAmp Primers Pool A or B (Applied Biosystems). The following cycling conditions were applied: denaturation for 10 minutes at 95°C, 1 step at 55°C for 2 minutes followed by 2 minutes at 72°C, and 12 cycles (95°C for 15 seconds, 60°C for 4 minutes). According to manufacturer’s instructions, the preamplified cDNA product was loaded onto TaqMan MicroRNA Array A or TaqMan MicroRNA Array B after mixing with water and TaqMan Universal PCR Master Mix, with Uracil-DNA glycosylase (UNG). The following real-time PCR protocol was used: 2 min at 50°C, 10 min at 95°C, 40 cycles of (30 s at 95°C and 1 min at 60°C). Real time PCR reactions were performed on an ABI ViiA 7 Real-Time PCR System (Applied Biosystems). The results were analyzed using ABI ViiA 7 Real-Time PCR software (Applied Biosystems), based on the comparative Ct method (delta deltaCt). The amplification signal was checked on each sample by ABI ViiA 7 Real-Time PCR System software. Data were normalized using a modified global mean normalization strategy based on common targets. The global mean on common targets strategy calculates normalization factors based on the geometric mean of the Relative Quantities (RQs) of all genes that are measured in all samples. It is a variant on the global mean normalization strategy and has proven to be the most accurate and sensitive approach to analyze high-throughput miRNA profiles [30], [31]. Analyses were restricted to miRNAs that were detectable in a minimum of 80% of samples (i.e. miRNAs that were expressed in at least 16 out of the 20 samples analyzed in this study) [32]; 289 miRNAs were chosen for subsequent analysis based on this threshold criterion.

### *In-vitro* analysis of IFN-α treatment effects

PBMCs were collected from eight healthy (HIV- and HCV-negative) donors. All donor samples are routinely tested for a comprehensive panel of bloodborne pathogens upon collection including HIV, HCV and HBV using ultrasensitive PCR (nucleic acid test yield) and serology. The healthy donor study protocols were approved by the UCSF Committee on Human Research. CD4+ T cells were isolated using bead-based negative selection (STEMCELL Technologies). Cells were plated at a million cells per well and treated with either 5 U/ml of IFN-α-A2 (R&D Systems) or media as a negative control. The expression of miR-422a was measured after 24 hours of stimulation using Taqman primers and probes (Life Technologies).

### Quantitative PCR measurement of MLH1 and TP53 mRNA expression

RNA from PBMCs was transcribed into cDNA using the SuperScript VILO cDNA Synthesis Kit (Invitrogen). Quantitative real-time PCR measuring MLH1 and TP53 using Taqman real time PCR was performed using the ABI ViiA 7 Real-Time PCR System. Raw cycle threshold (Ct) numbers of amplified gene products were normalized to the housekeeping gene ribosomal protein, large, P0 (RPLP0) to control for cDNA input amounts. RPLP0 was chosen as the housekeeping gene based on our previous analyses of the same set of samples [16]. We previously tested a panel of six housekeeping genes (GAPDH, 18S, ACTB, PPIA, RPLP0, and UBC). The GeNorm algorithm [33] identified RPLP0 as the most stably expressed housekeeping gene. Fold induction was determined using the comparative Ct method [33].

### Statistical analysis

We identified differentially expressed miRNAs between pre-, during, and post-IFN-α/RBV time points using paired t-tests for each miRNA. To adjust for multiple comparisons, false discovery rates (FDR) were computed using the Benjamini-Hochberg procedure [34]. Viral load values were log_10_ transformed, and miRNA values were global-normalized and then log_10_ transformed. The missing values for each miRNA were imputed by the minimum detected value minus 0.5. After log_10_ transformation and imputation, the within-group standard deviations (median across microRNAs) were 0.79 for peg-IFN-α/RBV timepoints, and 0.88 for during-IFN-α/RBV timepoints.

### miRNA interactome characterization

Lists of restriction factors and miRNAs that were modulated by exogenous IFN-α/RBV treatment were uploaded to the Ingenuity Pathway Analysis (IPA) tool (Ingenuity Systems, www.ingenuity.com), and were analyzed based on the IPA library of canonical pathways. IPA was implemented to create a genetic interaction network depicting known experimentally validated relationships.

### miRNA-mRNA network inference

We used two variables to generate a network between miRNAs and anti-HIV-1 restriction factor mRNAs: 1) inverse expression relationships between a given miRNA-mRNA pair, and 2) significant sequence homology between a given miRNA seed region and a restriction factor 3′ UTR. miRNA-mRNA inverse expression relationships were determined using the Pearson correlation coefficient (p-value <0.05, rho≤0.07). miRNA-mRNA sequence homology was determined by using blastn (http://blast.ncbi.nlm.nih.gov/) with word size 4, alignment length ≥5, and no mismatches allowed. We required reverse-complementing matches between miRNAs and mRNAs, with E-value cutoff ≤1.

## Results and Discussion

**Figure 1:**
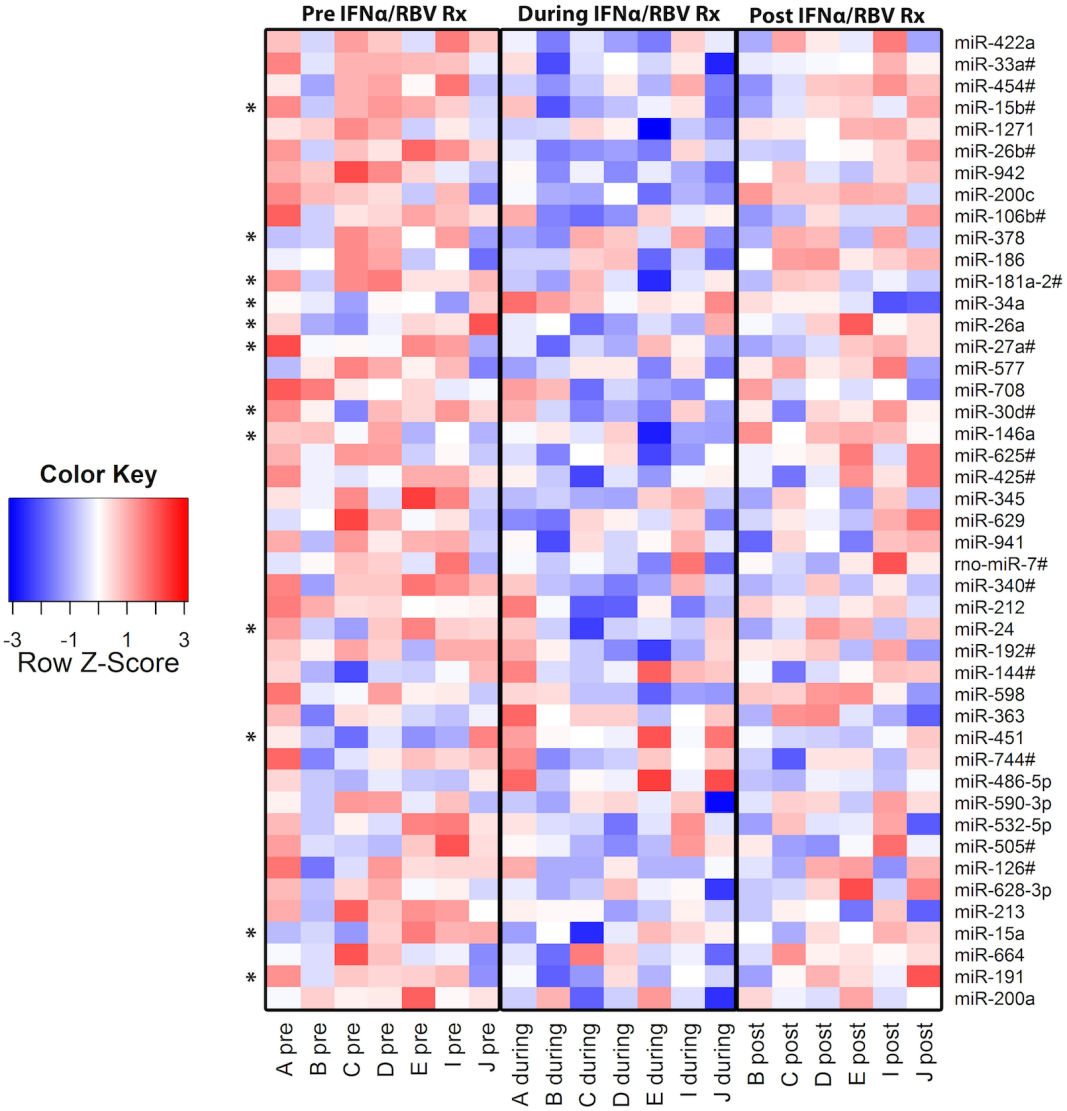
Heat map representing effects of IFN-α/RBV treatment on the expression of individual miRNAs. 45 miRNAs were significantly modulated by IFN-α/RBV *in vivo*, based on a paired t test and an uncorrected significance cutoff of p<0.05. Relative copy numbers of each miRNA are reported. Blue color indicates ≤−3 SD from the mean, red indicates ≥3 SD from the mean, and white represents the mean. Asterisks indicate previously established association with HIV-1.

**Figure 2:**
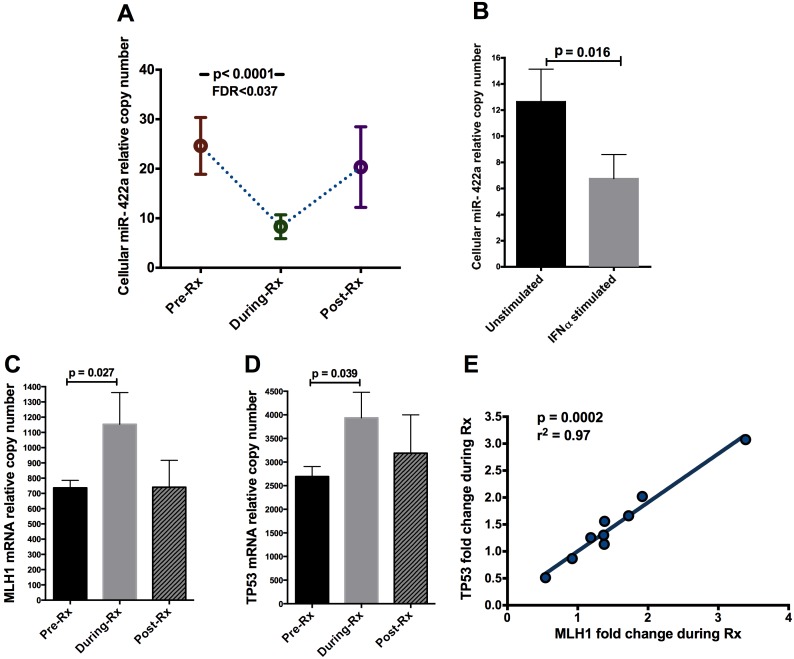
miR-422a is modulated by IFN-α treatment. (A) Expression of miR-422a in PBMC *in vivo* before, during and after IFN-α/RBV treatment (labeled as “Rx”). Error bars represent SEM. P-value was obtained using a paired t-test and FDR is reported. (B) Effects of IFN-α on the expression of miR-422a in CD4+ T cells *in vitro*; cells were plated at a million cells per well and treated with either 5 U/ml of IFN-α or media as a negative control. (C) Expression of MLH1 in PBMC *in vivo* before, during, and after IFN-α/RBV treatment. (D) Expression of TP53 in PBMC *in vivo* before, during, and after IFN-α/RBV treatment. Error bars represent SEM. P-values were obtained using paired Wilcoxon tests. (E) Correlation between TP53 fold induction and MLH1 fold induction in PBMC *in vivo* during IFN-α/RBV treatment. P-value was obtained using Spearman’s rank test.

We examined the effects of exogenous IFN-α treatment on PBMC miRNA profile, focusing on seven subjects before, during and after IFN-α/RBV therapy (Table S1). A total of 754 established miRNA targets were surveyed in PBMCs. Based on our threshold criterion (detectable expression in a minimum of 80% of samples), 289 miRNAs were chosen for subsequent analysis. We aimed to identify particular miRNA variants that were up- or down-regulated in PBMCs during IFN-α/RBV treatment consistently across individuals. IFN-α/RBV did not significantly affect expression levels of miRNA machinery genes (Fig. S1) suggesting that observed effects of IFN-α/RBV on particular miRNAs were specific in nature rather than the consequence of nonspecific shifts in global miRNA production. Of all 754 miRNAs measured, 45 miRNAs were significantly modulated by IFN-α/RBV *in vivo*, based on a paired t test and an uncorrected significance cutoff of p<0.05 (Fig. 1, Table S2). Twelve miRNAs in this list have been previously associated with HIV-1 infection (Table S2). However, due to the large number of miRNAs surveyed, accounting for multiple comparisons is imperative. After correcting for false discovery rate to account for multiple comparisons, only one miRNA out of the 45 initially identified, miR-422a, was significantly modulated by IFN-α/RBV *in vivo* using our highly stringent statistical criteria (p<0.0001, paired t test; FDR<0.037) (Fig. 2A). We then performed a controlled *in vitro* experiment in the absence of ribavirin co-administration and HIV or HCV infection to determine if IFN-α treatment suppresses miR-422a expression in CD4+ T cells, the principal target cells of HIV-1 infection in peripheral tissues. This experiment was conducted to evaluate the possibility that miR-422a modulation by IFN-α occurs within the target cell and may therefore play a role in determining cell-intrinsic susceptibility to HIV-1 infection. CD4+ T cells were negatively selected from fresh peripheral blood collected from eight HIV- and HCV-uninfected donors and treated with IFN-α *in vitro*. IFN-α treatment significantly suppressed miR-422a in CD4+ T cells *in vitro* (p = 0.016), extending the primary result of our *in vivo* expression profiling experiment (Fig. 2B).

We next sought to determine if any miRNAs exhibited significant correlations with HIV-1 viral load in our study population. Results are presented in Table 1. Several of the miRNAs exhibiting correlations with plasma viremia have been previously associated with HIV-1. The copy numbers of seven miRNAs were significantly correlated with baseline, pre-IFN-α/RBV HIV-1 viral load (p<0.05, Spearman’s rank): miR-29a, miR-101, miR-195, miR-25#, miR-491, miR-503, and miR-885. The copy numbers of six miRNAs were correlated with HIV-1 viral load during IFN-α/RBV: miR-138, miR-Let-7e, miR-10a, miR-145#, miR-31, and miR-589. Lastly, log HIV-1 viral load reduction during IFN-α/RBV therapy was significantly correlated with fold change of the following four miRNAs: miR-30e-3p, miR-148b#, miR-30d, and miR-589 (IFN-α/RBV treatment reduced plasma viral load by 0.80 (±0.33) log_10_ copies/ml during treatment, and viremia typically returned to approximate pre-treatment levels following therapy). Of particular relevance in these lists, miR-29a and miR-138 have been demonstrated to target HIV-1 directly [35], and both miRNAs demonstrated a negative correlation with HIV viral load in the current study.

**Table 1:** Correlations between miRNA and HIV-1 viral load.

microRNA	Spearman r	p value	R square	Previously associated with HIV? Y/N [ref]
**Correlations between pre-IFN-α/RBV miRNA relative copy number and HIV-1 viral load.**				
miR-25#	0.919	0.007	0.393	N
miR-885-5p	0.857	0.024	0.888	N
miR-195	−0.821	0.034	0.426	Y [55]
miR-29a	−0.786	0.048	0.635	Y [56], [57]
miR-101	−0.786	0.048	0.244	Y [58]
miR-503	−0.786	0.048	0.210	Y [59]
miR-491-5p	−0.786	0.048	0.191	N
**Correlations between miRNA relative copy number and HIV-1 viral load during IFN-α/RBV.**				
miR-145#	0.883	0.015	0.696	N
miR-138	−0.857	0.024	0.746	Y [35]
miR-10a	−0.821	0.034	0.406	N
miR-31	−0.786	0.048	0.755	Y [59], [60]
miR-589	−0.786	0.048	0.536	N
let-7e	−0.786	0.048	0.507	Y [61]
**Correlations between miRNA expression fold change and HIV-1 log viral load reduction.**				
miR-30e-3p	0.821	0.034	0.522	Y [62]
miR-148b#	0.786	0.048	0.547	Y [63]
miR-30d	0.786	0.048	0.488	Y [55]
miR-589	0.786	0.048	0.431	N

Based on our data suggesting that miR-422a is the only miRNA that is significantly modulated by IFN-α *in vivo* in PBMCs, we performed a comprehensive search of the literature to identify established gene targets that are known to be regulated by miR-422a. To the best of our knowledge, there are only two experimentally verified regulatory targets of miR-422a: CYP7A1, a gene involved in regulation of bile acid synthesis in the liver [36], and MLH1, a gene involved in DNA mismatch repair that is a MutLα component [37]. According to a study by Mao et al [37], miR-422a negatively regulates the expression of MLH1. Overexpression of MLH1 induces apoptosis by blocking transcription on damaged DNA templates, resulting in p53 induction [38], [39]. The IFN-α-mediated suppression of miR-422a observed in our preliminary experiments would be expected to result in increased expression of MLH1, which in turn should elevate p53 expression, resulting in the induction of apoptosis. We sought to confirm these predicted relationships using our existing PBMC specimens from the seven SHCS patients, by measuring the mRNA expression of MLH1 and TP53 (the gene encoding p53) before and during IFN-α/RBV treatment. As predicted, both MLH1 and TP53 expression were elevated during the IFN-α/RBV treatment period (Fig. 2C and 2D), and a near perfect correlation was observed between the induction of the two genes (Fig. 2E). Although this does not represent direct evidence of a regulatory relationship between miR-422a and MLH1 (and TP53), the observed expression patterns are compatible with this regulatory scenario and consistent with the published literature.

**Figure 3:**
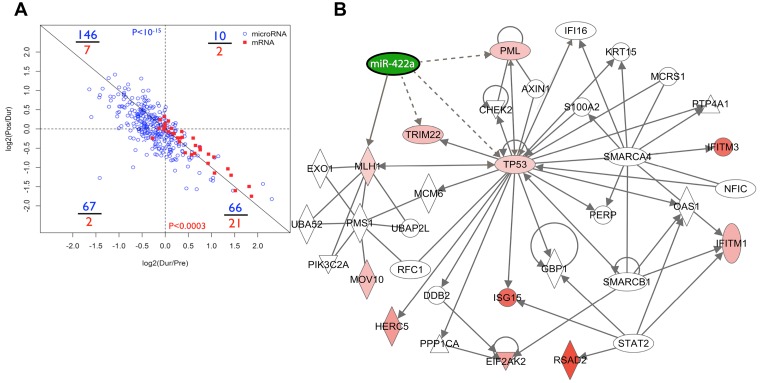
Visualization of miRNA and mRNA regulatory networks. (A) Plot of global miRNA and anti-HIV-1 restriction factor responses to IFN-α/RBV treatment. Numbers in blue and red represent tallies of microRNAs and restriction factor mRNAs in each quadrant, respectively. P-values were obtained using Fisher’s exact tests. (B) Integrative bioinformatic analyses of our miRNA data, restriction factor, MLH1 and TP53 mRNA profiles within the context of gene regulatory networks. Ingenuity Pathway Analysis (IPA) software was implemented to create the network map.

We aimed to expand our understanding of the miR-422a interactome beyond direct experimentally-validated relationships using a combination of experimental and bioinformatic techniques. We hypothesized that IFN-α-modulated miRNAs suppress HIV-1 replication by enhancing the expression of anti-HIV-1 host restriction factors. We recently characterized the mRNA expression of all established anti-HIV-1 host restriction factors in our collection of longitudinal SHCS samples, demonstrating that IFN-α treatment significantly induces several restriction factors *in vivo* [15], [16]. We exploited our previously generated gene expression data to simultaneously visualize IFN-α effects on miRNA and restriction factor mRNA profiles *in vivo*. Our visualization demonstrated that global miRNA expression was typically suppressed by IFN-α treatment, while restriction factors were typically induced (Fig. 3A). The distribution of data was highly and significantly skewed; miRNAs predominantly occupied the upper-left quadrant (representing suppression during IFN-α treatment and rebound to approximate baseline levels post-treatment), while restriction factor mRNAs predominantly occupied the lower-right quadrant (representing induction during IFN-α treatment and rebound to approximate baseline levels post-treatment). Although these correlative data do not prove that negative regulatory relationships exist between miRNAs and restriction factor mRNAs, the observed pattern is compatible, provocative, and worthy of further consideration.

We next implemented Ingenuity Pathway Analysis (IPA) software to perform integrative bioinformatic analyses of our miRNA, restriction factor, MLH1 and TP53 mRNA profiles within the context of gene regulatory networks. By merging our expression data from SHCS subjects with the Ingenuity Knowledge Base, IPA assembled a rich genetic interaction network depicting known experimentally validated relationships (Fig. 3B). There are a few prominent features within the IPA network that warrant mention. First, in addition to the previously known relationship with MLH1, miR-422a is predicted to directly regulate TP53 and the tripartite motif (TRIM) family anti-HIV-1 restriction factors TRIM19 (PML) and TRIM22. This prediction is based on sequence homology between the miR-422a seed region sequence and the 3′ UTR of TP53, TRIM19 (PML) and TRIM22. In alignment with this prediction, both TRIM19 (PML) and TRIM22 are induced by IFN-α/RBV according to our published data [16]. In addition to putative antiviral functions, TRIM19 (PML) is a potent driver of DNA damage-induced apoptosis, and physically interacts with p53 *in vitro* and *in vivo.* PML acts as a transcriptional co-activator with p53 and potentiates the antiproliferative downstream effects of p53 [40]. Second, TP53 is the epicenter of the miR-422a genetic interaction network, exhibiting numerous direct and indirect connections with host restriction factors. Accordingly, IFN-α has been shown to enhance the transcription of p53 target genes and p53-dependent apoptosis, and can directly induce expression of p53 [41]. Lastly, the gamma interferon-inducible gene IFI16 appears in the network. IFI16 has recently been characterized as a DNA sensor that plays a critical role in triggering the caspase 1-mediated pyroptosis of abortively-infected cells [42], which may be a principal mechanism underlying CD4 T cell depletion in HIV-1-infected individuals [43]. Taken together, the IFN-α-suppressed miRNA miR-422a is embedded in a rich interactome associated with both control of viral replication and apoptotic induction.

**Figure 4:**
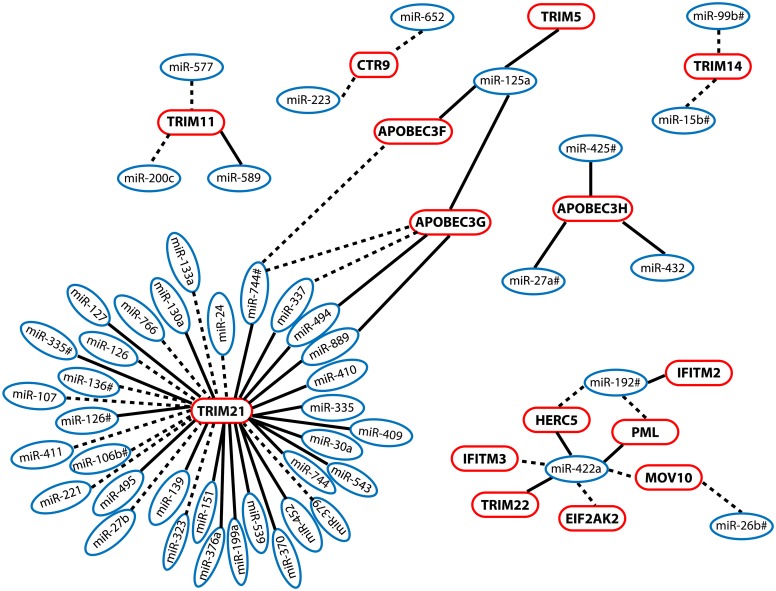
Global network of putative regulatory interactions between miRNAs and anti-HIV-1 restriction factor mRNAs. miRNA and mRNA expression data were measured in longitudinal PBMC samples from IFN-α/RBV-treated patients enrolled in the SHCS. Two variables were used to generate the network: 1) inverse expression relationships between a given miRNA-mRNA pair (represented as a dashed line drawn between a miRNA – mRNA pair), and 2) Significant sequence homology between a given miRNA seed region and a restriction factor 3′ UTR (word size 4, alignment length > = 5, e-value<1.0). Significant sequence homology is represented by a solid line. miRNAs are listed in blue; restriction factors are listed in pink.

To complement our focused analyses of miR-422a, we developed and implemented a computational approach to infer regulatory networks between the entire repertoire of surveyed miRNAs and the mRNA expression of anti-HIV-1 restriction factors observed in our IFN-α/RBV-treated SHCS subjects. Our approach is derived from a similar experiment used to examine miRNA-mRNA pairs within the context of HCV infection [44]. We used two variables to generate the network: 1) inverse expression relationships between a given miRNA-mRNA pair, and 2) significant sequence homology between a given miRNA seed region and a restriction factor 3′ UTR (word size 4, alignment length > = 5, e-value<1.0). These relationships were interpreted as putative negative regulatory interactions between a miRNA and mRNA. Our approach revealed a large number of potential regulatory interactions between miRNAs and restriction factor mRNAs (Fig. 4); 15 out of the 34 restriction factors that were up-modulated in SHCS patients undergoing IFN-α/RBV treatment were associated with at least 1 putative regulatory miRNA, and 29 distinct miRNAs were involved in these predicted relationships. To evaluate the likelihood that our network inference strategy was revealing legitimate regulatory relationships, we compared the frequencies of significant miRNA seed sequence – mRNA 3′ UTR homology hits between miRNA – mRNA pairs with significant inverse expression relationships (35 out of 62) and pairs without inverse expression correlations (8455 out of 25,636). Using a Fisher’s Exact test, we were able to determine that miRNA-mRNA sequence homology was observed at a significantly higher frequency in inverse expression relationships (p = 8*10^−6^, OR = 3.2 [95% CI 1.9, 5.6]), implying that the identified miRNA-mRNA networks might in fact play a role in regulating restriction factor expression.

Our analyses revealed a provocative list of 22 distinct potential miRNA regulators of TRIM21. The TRIM family contains over 60 proteins and exhibits a wide range of activities, including viral suppression and regulation of innate and adaptive immune responses. TRIM21 is known to play a crucial role in regulating type I interferon production [45]. Recently, TRIM21 was reported to recognize and degrade viruses in the cytoplasm by binding to antibody-coated virions [46]–[48]. Therefore, TRIM21 acts as an intracellular arm of adaptive immunity, and serves as a direct link between cell-intrinsic and adaptive immune processes. Our list of predicted miRNA regulators of TRIM21 expression warrants further investigation and may be exploited to enhance the potent antiviral activity of TRIM21 *in vivo*.

Our collection of clinical samples does not allow us to evaluate the possibility that the inclusion of ribavirin in anti-HCV therapy and universal HCV-coinfection in our cohort may affect our findings. However, ribavirin treatment has been previously shown to have negligible effects against HIV-1 (56). Moreover, gene expression profiles of HIV-1-monoinfected individuals undergoing IFN-α monotherapy [5], [49] match our gene expression data from IFN-α/RBV-treated HIV/HCV-coinfected patients [15], [16]. We confirmed the IFN-α-mediated suppression of miR-422a by performing an *in vitro* experiment in isolated peripheral blood CD4+ T cells in the absence of ribavirin and HCV infection, which extends our *in vivo* observations in IFN-α/RBV-treated HIV/HCV-coinfected individuals. Although our principal result involving miR-422a is not likely to have been affected by ribavirin administration or HCV coinfection, future miRNA profiling studies of HIV-1-monoinfected individuals undergoing IFN-α monotherapy will complement our observations reported here.

Amongst the HIV/HCV-coinfected participants enrolled in the Swiss HIV Cohort Study, ∼85% report intravenous drug use. In regards to potential confounding effects of IDU status on our findings, morphine usage may affect HIV disease outcomes and accelerate disease progression [50], [51]. Moreover, *in vitro* studies have demonstrated that morphine treatment increases cellular susceptibility to HIV infection, inhibits production of IFN-α and IFN-β antiviral cytokines [52], and modulates the expression of anti-HIV-1 miRNAs (miRNA-28, 125b, 150, and 382) [53]. Importantly, none of these opioid-modulated miRNAs were modulated in response to IFN-α/RBV therapy in our study.

In summary, our data demonstrate that a specific reduction of cellular miR-422a is associated with the suppression of HIV-1 by IFN-α *in vivo*. Our tiered network analyses suggest that miR-422a may contribute to the IFN-α-mediated suppression of HIV-1 viremia and decay of the latent reservoir via regulation of multiple retroviral restriction factors and genes involved in p53-dependent apoptosis and pyroptosis pathways. In addition, our global miRNA surveys identified several miRNAs whose expression levels were significantly correlated with HIV-1 viral load, and 29 distinct miRNAs that may regulate anti-HIV-1 restriction factor expression *in vivo*. Comprehensive miRNA profiling of isolated cellular subsets (e.g. CD4+ T cells, CD8+ T cells, B cells and monocytes) in subsequent studies will complement these observations. The possibility exists that one or more of these identified miRNAs may be manipulated to control HIV-1. *In vivo* targeting of the liver-specific miRNA and essential HCV cofactor miR-122 by the small molecule drug “miravirsen” achieves up to a three-log reduction in HCV viral load which persists indefinitely after treatment cessation [54]. This provides a promising model, and suggests that the development and deployment of miRNA-based therapeutic strategies for other chronic viral pathogens including HIV-1 is likely achievable.

To the best of our knowledge, our study is the first to demonstrate the effect of IFN-α treatment on miRNA expression profile *in vivo*, and these are the first data associating miR-422a with HIV-1 infection. Future work should validate and extend the translational and *in silico* observations reported here with detailed *in vitro* analyses of miR-422a effects on HIV-1 replication and the lifespan of HIV-1-infected cells.

## Supporting Information

Figure S1 **IFN-α/RBV treatment does not alter the expression of microRNA machinery genes** ***in vivo*.** Expression of (A) DROSHA, (B) DICER1, and (C) DGCR8 miRNA machinery genes before, during, and after IFN-α/RBV treatment. Black, light grey, and dark grey bars represent pre-treatment, during treatment, and post-treatment expression levels, respectively. P-values were obtained using paired Wilcoxon tests.
(PDF)

Table S1 **SHCS subject characteristics at baseline (pre IFN-α/RBV treatment).**
(PDF)

Table S2 **List of microRNAs modulated by IFN-α/RBV with p-value<0.05 (pre Rx/during Rx).**
(PDF)

## References

[1] Isaacs A, Lindenmann J (1957) Virus interference. I. The interferon. Proc R Soc Lond B Biol Sci 147: 258–267. 26297790

[2] Stetson DB, Medzhitov R (2006) Type I interferons in host defense. Immunity 25: 373–381. 16979569 10.1016/j.immuni.2006.08.007

[3] Marucco DA, Veronese L, de Requena DG, Bonora S, Calcagno A, et al (2007) Antiretroviral activity of pegylated interferon alfa-2a in patients co-infected with HIV/hepatitis C virus. J Antimicrob Chemother 59: 565–568. 17213263 10.1093/jac/dkl497

[4] Torriani FJ, Rodriguez-Torres M, Rockstroh JK, Lissen E, Gonzalez-Garcia J, et al (2004) Peginterferon Alfa-2a plus ribavirin for chronic hepatitis C virus infection in HIV-infected patients. N Engl J Med 351: 438–450. 15282351 10.1056/NEJMoa040842

[5] Asmuth DM, Murphy RL, Rosenkranz SL, Lertora JJ, Kottilil S, et al (2010) Safety, tolerability, and mechanisms of antiretroviral activity of pegylated interferon Alfa-2a in HIV-1-monoinfected participants: a phase II clinical trial. J Infect Dis 201: 1686–1696. 20420510 10.1086/652420 PMC2946345

[6] Pillai S, Abdel-Mohsen M, Guatelli J, Skasko M, Monto A, et al. Interferon-α treatment potently suppresses HIV-1 viremia by inducing host restriction factors in vivo. (submitted).

[7] Azzoni L, Foulkes AS, Papasavvas E, Mexas AM, Lynn KM, et al (2013) Pegylated Interferon Alfa-2a Monotherapy Results in Suppression of HIV Type 1 Replication and Decreased Cell-Associated HIV DNA Integration. J Infect Dis 207: 213–222. 23105144 10.1093/infdis/jis663 PMC3532820

[8] McNamara LA, Collins KL (2013) Interferon Alfa Therapy: Toward an Improved Treatment for HIV Infection. J Infect Dis 207: 201–203. 23105145 10.1093/infdis/jis667

[9] Sun H, Buzon MJ, Shaw A, Berg RK, Yu XG, et al. (2013) Hepatitis C Therapy With Interferon-alpha and Ribavirin Reduces CD4 T-Cell-Associated HIV-1 DNA in HIV-1/Hepatitis C Virus-Coinfected Patients. J Infect Dis. 10.1093/infdis/jit628 PMC3982848 24277743

[10] Rempel H, Sun B, Calosing C, Pillai SK, Pulliam L (2010) Interferon-alpha drives monocyte gene expression in chronic unsuppressed HIV-1 infection. AIDS 24: 1415–1423. 20495440 10.1097/QAD.0b013e32833ac623 PMC2991092

[11] Rotger M, Dang KK, Fellay J, Heinzen EL, Feng S, et al (2010) Genome-wide mRNA expression correlates of viral control in CD4+ T-cells from HIV-1-infected individuals. PLoS Pathog 6: e1000781. 20195503 10.1371/journal.ppat.1000781 PMC2829051

[12] Teijaro JR, Ng C, Lee AM, Sullivan BM, Sheehan KC, et al (2013) Persistent LCMV infection is controlled by blockade of type I interferon signaling. Science 340: 207–211. 23580529 10.1126/science.1235214 PMC3640797

[13] Wilson EB, Yamada DH, Elsaesser H, Herskovitz J, Deng J, et al (2013) Blockade of chronic type I interferon signaling to control persistent LCMV infection. Science 340: 202–207. 23580528 10.1126/science.1235208 PMC3704950

[14] Portales P, Reynes J, Pinet V, Rouzier-Panis R, Baillat V, et al (2003) Interferon-alpha restores HIV-induced alteration of natural killer cell perforin expression in vivo. AIDS 17: 495–504. 12598769 10.1097/00002030-200303070-00004

[15] Pillai SK, Abdel-Mohsen M, Guatelli J, Skasko M, Monto A, et al (2012) Role of retroviral restriction factors in the interferon-alpha-mediated suppression of HIV-1 in vivo. Proc Natl Acad Sci U S A 109: 3035–3040. 22315404 10.1073/pnas.1111573109 PMC3286922

[16] Abdel-Mohsen M, Deng X, Liegler T, Guatelli JC, Salama MS, et al (2014) Effects of Alpha Interferon Treatment on Intrinsic Anti-HIV-1 Immunity In Vivo. J Virol 88: 763–767. 24155399 10.1128/JVI.02687-13 PMC3911728

[17] Abdel-Mohsen M, Raposo RA, Deng X, Li M, Liegler T, et al (2013) Expression profile of host restriction factors in HIV-1 elite controllers. Retrovirology 10: 106. 24131498 10.1186/1742-4690-10-106 PMC3827935

[18] Bartel DP (2004) MicroRNAs: genomics, biogenesis, mechanism, and function. Cell 116: 281–297. 14744438 10.1016/s0092-8674(04)00045-5

[19] Poy MN, Spranger M, Stoffel M (2007) microRNAs and the regulation of glucose and lipid metabolism. Diabetes Obes Metab 9 Suppl 2: 67–73. 17919180 10.1111/j.1463-1326.2007.00775.x

[20] Calin GA, Croce CM (2006) MicroRNA-cancer connection: the beginning of a new tale. Cancer Res 66: 7390–7394. 16885332 10.1158/0008-5472.CAN-06-0800

[21] Scaria V, Hariharan M, Maiti S, Pillai B, Brahmachari SK (2006) Host-virus interaction: a new role for microRNAs. Retrovirology 3: 68. 17032463 10.1186/1742-4690-3-68 PMC1626483

[22] Althaus CF, Vongrad V, Niederost B, Joos B, Di Giallonardo F, et al (2012) Tailored enrichment strategy detects low abundant small noncoding RNAs in HIV-1 infected cells. Retrovirology 9: 27. 22458358 10.1186/1742-4690-9-27 PMC3341194

[23] Schopman NC, Willemsen M, Liu YP, Bradley T, van Kampen A, et al (2012) Deep sequencing of virus-infected cells reveals HIV-encoded small RNAs. Nucleic Acids Res 40: 414–427. 21911362 10.1093/nar/gkr719 PMC3245934

[24] Kozomara A, Griffiths-Jones S (2011) miRBase: integrating microRNA annotation and deep-sequencing data. Nucleic Acids Res 39: D152–157. 21037258 10.1093/nar/gkq1027 PMC3013655

[25] Huang J, Wang F, Argyris E, Chen K, Liang Z, et al (2007) Cellular microRNAs contribute to HIV-1 latency in resting primary CD4+ T lymphocytes. Nat Med 13: 1241–1247. 17906637 10.1038/nm1639

[26] Wang X, Ye L, Hou W, Zhou Y, Wang YJ, et al (2009) Cellular microRNA expression correlates with susceptibility of monocytes/macrophages to HIV-1 infection. Blood 113: 671–674. 19015395 10.1182/blood-2008-09-175000 PMC2628373

[27] Kulkarni S, Savan R, Qi Y, Gao X, Yuki Y, et al (2011) Differential microRNA regulation of HLA-C expression and its association with HIV control. Nature 472: 495–498. 21499264 10.1038/nature09914 PMC3084326

[28] Pedersen IM, Cheng G, Wieland S, Volinia S, Croce CM, et al (2007) Interferon modulation of cellular microRNAs as an antiviral mechanism. Nature 449: 919–922. 17943132 10.1038/nature06205 PMC2748825

[29] Schoeni-Affolter F, Ledergerber B, Rickenbach M, Rudin C, Gunthard HF, et al (2010) Cohort profile: the Swiss HIV Cohort study. Int J Epidemiol 39: 1179–1189. 19948780 10.1093/ije/dyp321

[30] Mestdagh P, Van Vlierberghe P, De Weer A, Muth D, Westermann F, et al (2009) A novel and universal method for microRNA RT-qPCR data normalization. Genome Biol 10: R64. 19531210 10.1186/gb-2009-10-6-r64 PMC2718498

[31] D’Haene B, Mestdagh P, Hellemans J, Vandesompele J (2012) miRNA expression profiling: from reference genes to global mean normalization. Methods Mol Biol 822: 261–272. 22144205 10.1007/978-1-61779-427-8_18

[32] Witwer KW, Clements JE (2012) Evidence for miRNA expression differences of HIV-1-positive, treatment-naive patients and elite suppressors: a re-analysis. Blood 119: 6395–6396. 22745299 10.1182/blood-2012-02-412742 PMC3448566

[33] Vandesompele J, De Preter K, Pattyn F, Poppe B, Van Roy N, et al (2002) Accurate normalization of real-time quantitative RT-PCR data by geometric averaging of multiple internal control genes. Genome Biol 3: RESEARCH0034. 12184808 10.1186/gb-2002-3-7-research0034 PMC126239

[34] Benjamini Y, Drai D, Elmer G, Kafkafi N, Golani I (2001) Controlling the false discovery rate in behavior genetics research. Behav Brain Res 125: 279–284. 11682119 10.1016/s0166-4328(01)00297-2

[35] Houzet L, Klase Z, Yeung ML, Wu A, Le SY, et al (2012) The extent of sequence complementarity correlates with the potency of cellular miRNA-mediated restriction of HIV-1. Nucleic Acids Res 40: 11684–11696. 23042677 10.1093/nar/gks912 PMC3526334

[36] Song KH, Li T, Owsley E, Chiang JY (2010) A putative role of micro RNA in regulation of cholesterol 7alpha-hydroxylase expression in human hepatocytes. J Lipid Res 51: 2223–2233. 20351063 10.1194/jlr.M004531 PMC2903801

[37] Mao G, Lee S, Ortega J, Gu L, Li GM (2012) Modulation of microRNA processing by mismatch repair protein MutLalpha. Cell Res 22: 973–985. 22290424 10.1038/cr.2012.18 PMC3367530

[38] Zhang H, Richards B, Wilson T, Lloyd M, Cranston A, et al (1999) Apoptosis induced by overexpression of hMSH2 or hMLH1. Cancer Res 59: 3021–3027. 10397236

[39] Yanamadala S, Ljungman M (2003) Potential role of MLH1 in the induction of p53 and apoptosis by blocking transcription on damaged DNA templates. Mol Cancer Res 1: 747–754. 12939400

[40] Guo A, Salomoni P, Luo J, Shih A, Zhong S, et al (2000) The function of PML in p53-dependent apoptosis. Nat Cell Biol 2: 730–736. 11025664 10.1038/35036365

[41] Porta C, Hadj-Slimane R, Nejmeddine M, Pampin M, Tovey MG, et al (2005) Interferons alpha and gamma induce p53-dependent and p53-independent apoptosis, respectively. Oncogene 24: 605–615. 15580300 10.1038/sj.onc.1208204

[42] Monroe KM, Yang Z, Johnson JR, Geng X, Doitsh G, et al. (2013) IFI16 DNA Sensor Is Required for Death of Lymphoid CD4 T Cells Abortively Infected with HIV. Science. 10.1126/science.1243640 PMC3976200 24356113

[43] Doitsh G, Galloway NL, Geng X, Yang Z, Monroe KM, et al. (2013) Cell death by pyroptosis drives CD4 T-cell depletion in HIV-1 infection. Nature. 10.1038/nature12940 PMC4047036 24356306

[44] Peng X, Li Y, Walters KA, Rosenzweig ER, Lederer SL, et al (2009) Computational identification of hepatitis C virus associated microRNA-mRNA regulatory modules in human livers. BMC Genomics 10: 373. 19671175 10.1186/1471-2164-10-373 PMC2907698

[45] Yang K, Shi HX, Liu XY, Shan YF, Wei B, et al (2009) TRIM21 is essential to sustain IFN regulatory factor 3 activation during antiviral response. J Immunol 182: 3782–3792. 19265157 10.4049/jimmunol.0803126

[46] McEwan WA, Tam JC, Watkinson RE, Bidgood SR, Mallery DL, et al (2013) Intracellular antibody-bound pathogens stimulate immune signaling via the Fc receptor TRIM21. Nat Immunol 14: 327–336. 23455675 10.1038/ni.2548 PMC3672961

[47] McEwan WA, Hauler F, Williams CR, Bidgood SR, Mallery DL, et al (2012) Regulation of virus neutralization and the persistent fraction by TRIM21. J Virol 86: 8482–8491. 22647693 10.1128/JVI.00728-12 PMC3421726

[48] Mallery DL, McEwan WA, Bidgood SR, Towers GJ, Johnson CM, et al (2010) Antibodies mediate intracellular immunity through tripartite motif-containing 21 (TRIM21). Proc Natl Acad Sci U S A 107: 19985–19990. 21045130 10.1073/pnas.1014074107 PMC2993423

[49] Hubbard JJ, Greenwell-Wild T, Barrett L, Yang J, Lempicki RA, et al (2012) Host gene expression changes correlating with anti-HIV-1 effects in human subjects after treatment with peginterferon Alfa-2a. J Infect Dis 205: 1443–1447. 22454462 10.1093/infdis/jis211 PMC3324397

[50] Ronald PJ, Robertson JR, Elton RA (1994) Continued drug use and other cofactors for progression to AIDS among injecting drug users. AIDS 8: 339–343. 7913328 10.1097/00002030-199403000-00007

[51] Specter S (1994) Drugs of abuse and infectious diseases. J Fla Med Assoc 81: 485–487. 7964576

[52] Wang Y, Wang X, Ye L, Li J, Song L, et al (2012) Morphine suppresses IFN signaling pathway and enhances AIDS virus infection. PLoS One 7: e31167. 22359571 10.1371/journal.pone.0031167 PMC3281044

[53] Wang X, Ye L, Zhou Y, Liu MQ, Zhou DJ, et al (2011) Inhibition of anti-HIV microRNA expression: a mechanism for opioid-mediated enhancement of HIV infection of monocytes. Am J Pathol 178: 41–47. 21224041 10.1016/j.ajpath.2010.11.042 PMC3069926

[54] Janssen HL, Reesink HW, Lawitz EJ, Zeuzem S, Rodriguez-Torres M, et al (2013) Treatment of HCV infection by targeting microRNA. N Engl J Med 368: 1685–1694. 23534542 10.1056/NEJMoa1209026

[55] Holland B, Wong J, Li M, Rasheed S (2013) Identification of human microRNA-like sequences embedded within the protein-encoding genes of the human immunodeficiency virus. PLoS One 8: e58586. 23520522 10.1371/journal.pone.0058586 PMC3592801

[56] Hariharan M, Scaria V, Pillai B, Brahmachari SK (2005) Targets for human encoded microRNAs in HIV genes. Biochem Biophys Res Commun 337: 1214–1218. 16236258 10.1016/j.bbrc.2005.09.183

[57] Ahluwalia JK, Khan SZ, Soni K, Rawat P, Gupta A, et al (2008) Human cellular microRNA hsa-miR-29a interferes with viral nef protein expression and HIV-1 replication. Retrovirology 5: 117. 19102781 10.1186/1742-4690-5-117 PMC2635386

[58] Mishra R, Singh SK (2013) HIV-1 Tat C modulates expression of miRNA-101 to suppress VE-cadherin in human brain microvascular endothelial cells. J Neurosci 33: 5992–6000. 23554480 10.1523/JNEUROSCI.4796-12.2013 PMC6618916

[59] Zhang ZN, Xu JJ, Fu YJ, Liu J, Jiang YJ, et al (2013) Transcriptomic analysis of peripheral blood mononuclear cells in rapid progressors in early HIV infection identifies a signature closely correlated with disease progression. Clin Chem 59: 1175–1186. 23592504 10.1373/clinchem.2012.197335

[60] Witwer KW, Watson AK, Blankson JN, Clements JE (2012) Relationships of PBMC microRNA expression, plasma viral load, and CD4+ T-cell count in HIV-1-infected elite suppressors and viremic patients. Retrovirology 9: 5. 22240256 10.1186/1742-4690-9-5 PMC3292811

[61] Swaminathan S, Suzuki K, Seddiki N, Kaplan W, Cowley MJ, et al (2012) Differential regulation of the Let-7 family of microRNAs in CD4+ T cells alters IL-10 expression. J Immunol 188: 6238–6246. 22586040 10.4049/jimmunol.1101196

[62] Hayes AM, Qian S, Yu L, Boris-Lawrie K (2011) Tat RNA silencing suppressor activity contributes to perturbation of lymphocyte miRNA by HIV-1. Retrovirology 8: 36. 21569500 10.1186/1742-4690-8-36 PMC3120759

[63] Yeung ML, Bennasser Y, Myers TG, Jiang G, Benkirane M, et al (2005) Changes in microRNA expression profiles in HIV-1-transfected human cells. Retrovirology 2: 81. 16381609 10.1186/1742-4690-2-81 PMC1352379

