# Supplementary figures and images for: Role of MicroRNA Modulation in the Interferon-α/Ribavirin Suppression of HIV-1 In Vivo
Source: PLoS One. 2014 Oct 2;9(10):e109220. doi: 10.1371/journal.pone.0109220 (PMC4183579; doi:10.1371/journal.pone.0109220)

Figure S1.

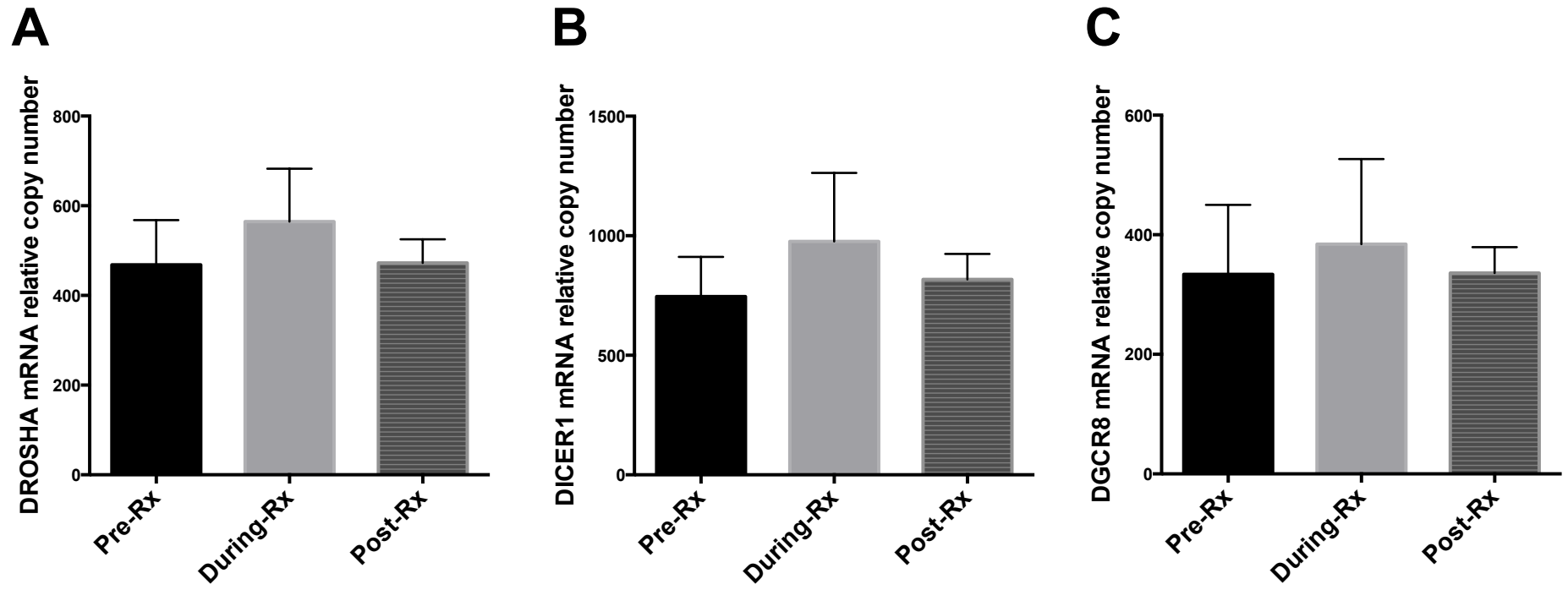

Supplement: Figure S1 — IFN-α/RBV treatment does not alter the expression of microRNA machinery genes in vivo. Expression of (A) DROSHA, (B) DICER1, and (C) DGCR8 miRNA machinery genes before, during, and after IFN-α/RBV treatment. Black, light grey, and dark grey bars represent pre-treatment, during treatment, and post-treatment expression levels, respectively. P-values were obtained using paired Wilcoxon tests. (PDF) [file pone.0109220.s001.pdf]
