# Supplementary material for: Role of MicroRNA Modulation in the Interferon-α/Ribavirin Suppression of HIV-1 In Vivo
Source: PLoS One. 2014 Oct 2;9(10):e109220. doi: 10.1371/journal.pone.0109220 (PMC4183579; doi:10.1371/journal.pone.0109220)
Supplement: Table S1 — SHCS subject characteristics at baseline (pre IFN-α/RBV treatment). (PDF) [file pone.0109220.s002.pdf]

Table S1. SHCS subject characteristics at baseline (pre IFN-α/RBV treatment).

Individual	Gender	Age	Risk group[1]	CDC disease stage[2]	Viral load pre-treatment (copies/ml)		Viral load during-treatment (copies/ml)		Viral load post-treatment (copies/ml)		CD4+ count (cells/µl)	HIV-1 subtype[3]	IFN-α/RBV sample[4]
					HIV-1	HCV	HIV-1	HCV	HIV-1	HCV			
A	M	38	IDU	A	43,200	500,000	11,400	0	N/A[5]	N/A	404	B	52.1
B	M	45	OTHER	B	55,215	2,490,000	6,322	2,980,000	29,857	N/A	270	B	13.6
C	M	42	MSM	A	78,886	9.9E+11	18,902	20	474,255	0	587	B	25.7
D	F	26	HET	A	13,100	700,000	10,986	0	15,072	461,701	374	B	48.6
E	M	28	HET	A	167,500	1,255,000	22,200	0	101,000	0	501	B	26.6
I	M	32	MSM	A	4,070	2,310,000	346	0	17,700	N/A	755	B	47.7
J	M	39	IDU	A	8,620	2,760,116	1,376	0	13,200	140,349	590	B	47.6

[1] Risk groups are defined as follows: HET = heterosexual, IDU = intravenous drug use, MSM = men who have sex with men.
[2] CDC disease stage is defined as follows: A = asymptomatic, acute infection or persistent generalized lymphadenopathy, B = symptomatic conditions, C = AIDS-indicator conditions.
[3] HIV-1 subtype was determined by applying the REGA subtyping algorithm to *pol* sequences (previously generated for drug resistance genotyping)
[4] Weeks post-initiation of IFN-α/RBV treatment that the on-treatment sample was collected.
[5] N/A = Not available
