# Supplementary material for: Role of MicroRNA Modulation in the Interferon-α/Ribavirin Suppression of HIV-1 In Vivo
Source: PLoS One. 2014 Oct 2;9(10):e109220. doi: 10.1371/journal.pone.0109220 (PMC4183579; doi:10.1371/journal.pone.0109220)
Supplement: Table S2 — List of microRNAs modulated by IFN-α/RBV with p-value<0.05 (pre Rx/during Rx). (PDF) [file pone.0109220.s003.pdf]

Table S2. List of microRNAs modulated by IFN-α/RBV with p value < 0.05 (pre Rx/during Rx).

	Log mean pre Rx	Log mean dur Rx	Log mean pos Rx	Log fold (pre Rx/during Rx)	p value (pre Rx/during Rx)	FDR value (pre Rx/during Rx)	Previously associated with HIV? Y/N [ref]
miR-422a	-12.27	-13.91	-13.21	1.64	0.0001	0.037	N
miR-33a#	-9.67	-10.85	-10.53	1.18	0.001	0.14	N
miR-454#	-10.03	-10.88	-10.39	0.85	0.002	0.14	N
miR-15b#	-5.67	-6.64	-6.37	0.97	0.002	0.14	Y [1]
miR-1271	-7.66	-8.89	-8.18	1.23	0.003	0.14	N
miR-26b#	-8.27	-9.70	-9.16	1.43	0.003	0.14	N
miR-942	-7.39	-7.99	-7.78	0.60	0.004	0.16	N
miR-200c	-5.87	-6.34	-5.94	0.47	0.01	0.19	N
miR-106b#	-6.69	-7.52	-7.61	0.83	0.01	0.19	N
miR-378	-7.52	-8.63	-8.53	1.12	0.01	0.19	Y [2]
miR-186	-0.98	-1.23	-0.89	0.25	0.01	0.19	N
miR-181a-2#	-7.76	-8.80	-8.82	1.04	0.01	0.19	Y [3]
miR-34a	-8.82	-7.79	-9.42	-1.03	0.01	0.19	Y [4]
miR-26a	-10.82	-11.83	-11.26	1.02	0.01	0.19	Y [5]
miR-27a#	-5.56	-6.56	-6.43	1.00	0.01	0.19	Y [6]
miR-577	-12.66	-13.75	-12.73	1.09	0.01	0.19	N
miR-708	-10.76	-12.36	-12.42	1.60	0.01	0.19	N
miR-30d#	-10.48	-11.67	-11.10	1.19	0.01	0.19	Y [6]
miR-146a	2.11	1.70	2.09	0.41	0.01	0.19	Y [7]
miR-625#	-4.47	-5.35	-4.70	0.87	0.01	0.19	N
miR-425#	-7.54	-8.90	-8.37	1.36	0.01	0.19	N

miR-345	-3.79	-4.38	-4.48	0.60	0.02	0.21	N
miR-629	-7.94	-8.46	-8.22	0.53	0.02	0.21	N
miR-941	-10.15	-11.16	-11.57	1.00	0.02	0.22	N
rno-miR-7#	-2.65	-3.16	-2.96	0.51	0.02	0.22	N
miR-340#	-6.20	-7.00	-7.08	0.80	0.02	0.23	N
miR-212	-9.18	-9.90	-9.66	0.72	0.02	0.23	N
miR-24	1.80	1.45	1.47	0.35	0.02	0.23	Y [6]
miR-378	-11.40	-12.04	-12.84	0.64	0.02	0.24	N
miR-192#	-12.44	-13.04	-12.90	0.60	0.03	0.24	N
miR-144#	-8.93	-6.81	-8.98	-2.11	0.03	0.24	N
miR-598	-8.02	-8.60	-8.20	0.58	0.03	0.26	N
miR-363	-9.26	-8.68	-9.56	-0.58	0.03	0.27	N
miR-451	-6.43	-4.12	-6.45	-2.30	0.03	0.27	Y [6]
miR-744#	-10.55	-10.79	-11.30	0.24	0.03	0.27	N
miR-486-5p	-8.32	-6.86	-8.61	-1.46	0.03	0.27	N
miR-590-3p	-6.93	-7.93	-7.57	1.00	0.04	0.27	N
miR-532-5p	-4.46	-4.90	-5.17	0.44	0.04	0.27	N
miR-505#	-10.26	-10.95	-11.09	0.69	0.04	0.27	N
miR-126#	-3.58	-4.46	-4.28	0.88	0.04	0.27	N
miR-628-3p	-9.47	-10.06	-9.68	0.59	0.04	0.27	N
miR-213	-8.78	-9.54	-9.98	0.76	0.04	0.27	N
miR-15a	-4.63	-5.18	-5.14	0.56	0.04	0.27	Y [5]
miR-664	-4.63	-5.12	-4.74	0.49	0.04	0.28	N
miR-191	2.37	1.89	2.15	0.48	0.04	0.28	Y [8]
miR-200a	-10.67	-11.57	-11.33	0.90	0.049	0.31	N

REFERENCES:

1. Shen CJ, Jia YH, Tian RR, Ding M, Zhang C, Wang JH. Translation of Pur-alpha is targeted by cellular miRNAs to modulate the differentiation-dependent susceptibility of monocytes to HIV-1 infection. *FASEB J* 2012,**26**:4755-4764.
2. Hariharan M, Scaria V, Pillai B, Brahmachari SK. Targets for human encoded microRNAs in HIV genes. *Biochem Biophys Res Commun* 2005,**337**:1214-1218.
3. Fujii YR, Saksena NK. Viral Infection-Related MicroRNAs in Viral and Host Genomic Evolution. In: *RNA and the Regulation of Gene Expression: A Hidden Layer of Complexity*. Edited by Morris KV: Caister Academic Press; 2008.
4. Zhang HS, Chen XY, Wu TC, Sang WW, Ruan Z. MiR-34a is involved in Tat-induced HIV-1 long terminal repeat (LTR) transactivation through the SIRT1/NFkappaB pathway. *FEBS Lett* 2012,**586**:4203-4207.
5. Sun G, Li H, Wu X, Covarrubias M, Scherer L, Meinking K*, et al.* Interplay between HIV-1 infection and host microRNAs. *Nucleic Acids Res* 2012,**40**:2181-2196.
6. Houzet L, Yeung ML, de Lame V, Desai D, Smith SM, Jeang KT. MicroRNA profile changes in human immunodeficiency virus type 1 (HIV-1) seropositive individuals. *Retrovirology* 2008,**5**:118.
7. Rom S, Rom I, Passiatore G, Pacifici M, Radhakrishnan S, Del Valle L*, et al.* CCL8/MCP-2 is a target for mir-146a in HIV-1-infected human microglial cells. *FASEB J* 2010,**24**:2292-2300.
8. Tan Gana NHT, Victoriano AF, Hibi Y, Asamitsu K, Uranishi H, Okamoto T. Interactions of cellular microRNAs in HIV-1 latently infected cells. In: *19th International AIDS Conference*. Washington, DC; 2012.
